# Effectiveness of Pre-procedural Mouth Rinses in Reducing Aerosol Contamination During Periodontal Prophylaxis: A Systematic Review

**DOI:** 10.3389/fmed.2021.600769

**Published:** 2021-06-10

**Authors:** Shahida Mohd-Said, Tuti Ningseh Mohd-Dom, Nawal Suhaimi, Haslina Rani, Colman McGrath

**Affiliations:** ^1^Faculty of Dentistry, Universiti Kebangsaan Malaysia (The National University of Malaysia), Kuala Lumpur, Malaysia; ^2^Faculty of Dentistry, The University of Hong Kong, Sai Ying Pun, Hong Kong

**Keywords:** COVID-19, periodontics, splatter, bioaerosol, air polishing, debridement, periodontal scaling

## Abstract

**Background:** Aerosol-producing dental procedures are of concern in the spread of infections, especially during the COVID-19 pandemic. Periodontal prophylaxis is the most common aerosol-producing procedure conducted in dental practice globally. During COVID-19, many national and international organizations advocated the use of pre-procedural mouth rinsing to prevent the spread of infections from aerosol-generating procedures in the dental setting; however, many questioned the scientific basis for such recommendations.

**Objective:** This systematic review aimed to evaluate the effectiveness of pre-procedural rinsing when preforming periodontal prophylaxis in reducing aerosol contamination in the dental setting.

**Methods:** A comprehensive standardized search strategy was employed, informed by a defined PICO question across four electronic databases. The review of the literature was conducted using the PRISMA framework. Agreement between assessors was determined throughout. Synthesis of study characteristics and key outcomes were conducted. Cochrane's risk-of-bias tool for randomized trials (RoB 2) was employed to assess the quality/bias among studies.

**Results:** The initial search yielded 731 citations across the four databases; 95 potentially effective studies were identified, with 56 effective studies found. Thirty randomized control trial studies were identified, 21 with a focus on effectiveness of pre-procedural mouth rinsing, involving 984 participants (aged 18–70). Agreement between assessors was high (Kappa >0.80). Various pre-procedural mouth rinses were tested, most frequently chlorhexidine (CHX) in 18 studies. The concentrations, volume, and prescribed duration of rinsing varied among studies, hampering meta-analyses. Nonetheless, all studies identified significant reductions in bacterial contamination, as measured by colony forming units (cfu). The effectiveness of CHX over other agents was evident with more than half of the studies (7/15) reporting over a 70% reduction in bacterial contamination (cfu). There were concerns over the risk of bias in most studies (76.2%); 19.0% had a high risk of bias and 4.8% were of low risk of bias.

**Conclusion:** There is substantial evidence to support pre-procedural mouth rinsing, such as with chlorohexidine, to effectively reduce aerosol contamination when performing periodontal prophylaxis compared to mouth rinsing with water or not rinsing.

## Introduction

The cornerstone for maintaining periodontal health is effective dental plaque control. This may be achieved through a combination of diligent home care practices and compliance to scheduled dental visits, whereby the dentist or hygienist undertakes the necessary scaling, polishing, and root debridement using ultrasonic scalers and air polishers. For individuals who have been affected with periodontitis, it is particularly crucial that they comply with scheduled dental visits regardless if they are in the active or maintenance phase of periodontal therapy.

Interruptions to regular dental services have been unexpectedly imposed due to the COVID-19 pandemic as part of the collective efforts to reduce the risk of transmission within dental clinics. Around the globe, the dental fraternity resolved that only urgent and emergency dental care should be permitted during the pandemic. Amidst dynamic health and community-related updates of the pandemic, the dental profession developed various guidelines to assist dentists to make appropriate clinical decisions in the management of their patients (1–3). These guidelines were necessary, although in some instances the available evidence to support them was questioned.

For patients with periodontal concerns, this disruption to scheduled periodontal therapy may not cause immediate pain or discomfort. Nonetheless, postponement of regular care can aggravate or be detrimental to their oral health status, may increase their risk of non-communicable diseases, or worsen their health status, particularly those with underlying systemic conditions (4). Non-surgical periodontal therapy like scaling, polishing, and root debridement are aerosol-generating procedures (AGP) and carry a high risk for aerosol contamination (5, 6). This causes concern for increased risk of infectious disease transmission during these procedures during the COVID-19 pandemic. There is an urgent need to reduce or eliminate the risk of aerosol contamination from AGPs given the necessity to carry out treatment to avoid periodontal disease progression (7).

One of the most widely advocated methods to reduce the level of contamination in the aerosol during dental procedures is pre-procedural mouth rinsing (8). The aim of our review was to investigate the effect of mouth rinsing before periodontal prophylaxis (pre-procedural mouth rinsing) on aerosol contamination in dental clinics. The findings from this review have implications in providing evidence to support or refute current guidelines for pre-procedural mouth rinsing.

## Methodology

### Search Strategy

The PICO strategy (9) was employed in focusing the review questions: What is the effectiveness of pre-procedural rinsing in periodontal prophylaxis in reducing aerosol contamination and what are the factors attributing to its effectiveness? The study population (**P**) was periodontitis patients receiving interventions (**I**) for reducing aerosol contaminations during non-surgical prophylaxis including dental scaling and tooth polishing, root planning or debridement, and air polishing using powered instruments with/without use of adjunctive antimicrobials. Findings from the relevant studies were to be compared (**C**) to the subjects or patients that did not receive similar interventions, with the primary outcomes (**O**) of interventions being reduced aerosol contamination.

### Selection Criteria

Electronic database searches were conducted on Scopus, MEDLINE via PubMed, Cochrane Library, and Web of Science up to 8th April 2020 using the predefined keywords “aerosol” and “dental prophylaxis” (Appendix 1 in Supplementary Material). Selection of key words and terms for search strategy were informed by Medical Subject Headings (MeSH) and previous related reviews (8, 10–19). No time limit was set in this search. Our initial search did not find any references for viral contamination in aerosol; hence the review is limited to bacterial contamination only.

### Data Selection and Extraction

The reviewers in this study were consulting specialists in Periodontology (S.M-S.) and Dental Public Health (T.N.M., H.R., and C.M.), and a dental graduate (N.S.). Initially, titles and abstracts were independently screened by two reviewers (S.M-S. and N.S.) to identify potential effective studies. Then, full-text articles were retrieved for the secondary screening by two additional reviewers (T.N.M., H.R., C.M.) for consensus on the eligibility. Disagreement between reviewers were resolved with the supervising author (C.M.) and Kappa statistics was used to assess the agreement between assessors throughout.

In synthesis of evidence from ‘effective’ studies, details included authors, article publication year, design of study, sampling size and allocation of test and control groups, details of intervention, type and description of periodontal prophylaxis procedures, and primary outcomes in terms of statistically significant findings and reduction of aerosol contamination between groups measured by colony forming units (cfu) using means and percentages. If permissible, mean cfu reduction % was calculated by Mean cfu reduction (%) = [(total amount of mean cfu at baseline—total amount of mean cfu after prophylaxis)/total amount of mean cfu at baseline] x 100%. When baseline data were not provided in the articles, mean reduction percentage was calculated as Mean cfu reduction (%) = (total amount of mean cfu for control group—total amount of mean cfu for test group)/total amount of mean cfu for control group] x 100%.

### Quality and Risk of Bias Assessment

An assessment of quality and risk of bias assessment was conducted on effective studies that informed the review employing the revised Cochrane risk-of-bias tool for randomized trials (RoB 2) (20). Domains evaluated were: Domain 1—risk of bias arising from the randomization process, Domain 2—risk of bias due to deviation from the intended interventions, Domain 3—risk of bias due to missing outcome data, Domain 4—risk of bias in measurement of outcomes, and Domain 5—risk of bias in the selection of the reposted results. Using specific signaling questions for each domain, response options including **Yes** (Y), **Probably yes** (PY), **Probably no** (PN), **No** (N), and **No information** (NI) available were made. Finally, the overall risk-of-bias judgement was made for each article based on the criteria for **Low Risk**, judged to raise **Some Concerns**, or **High Risk** of bias. Agreement between evaluators for this section (S.M-S., T.N.M., and H.R.) were discussed with the supervising author (C.M.) and the inter-evaluator reliability was calculated using Kappa statistics.

## Results

### Identification and Screening

**Figure 1:**
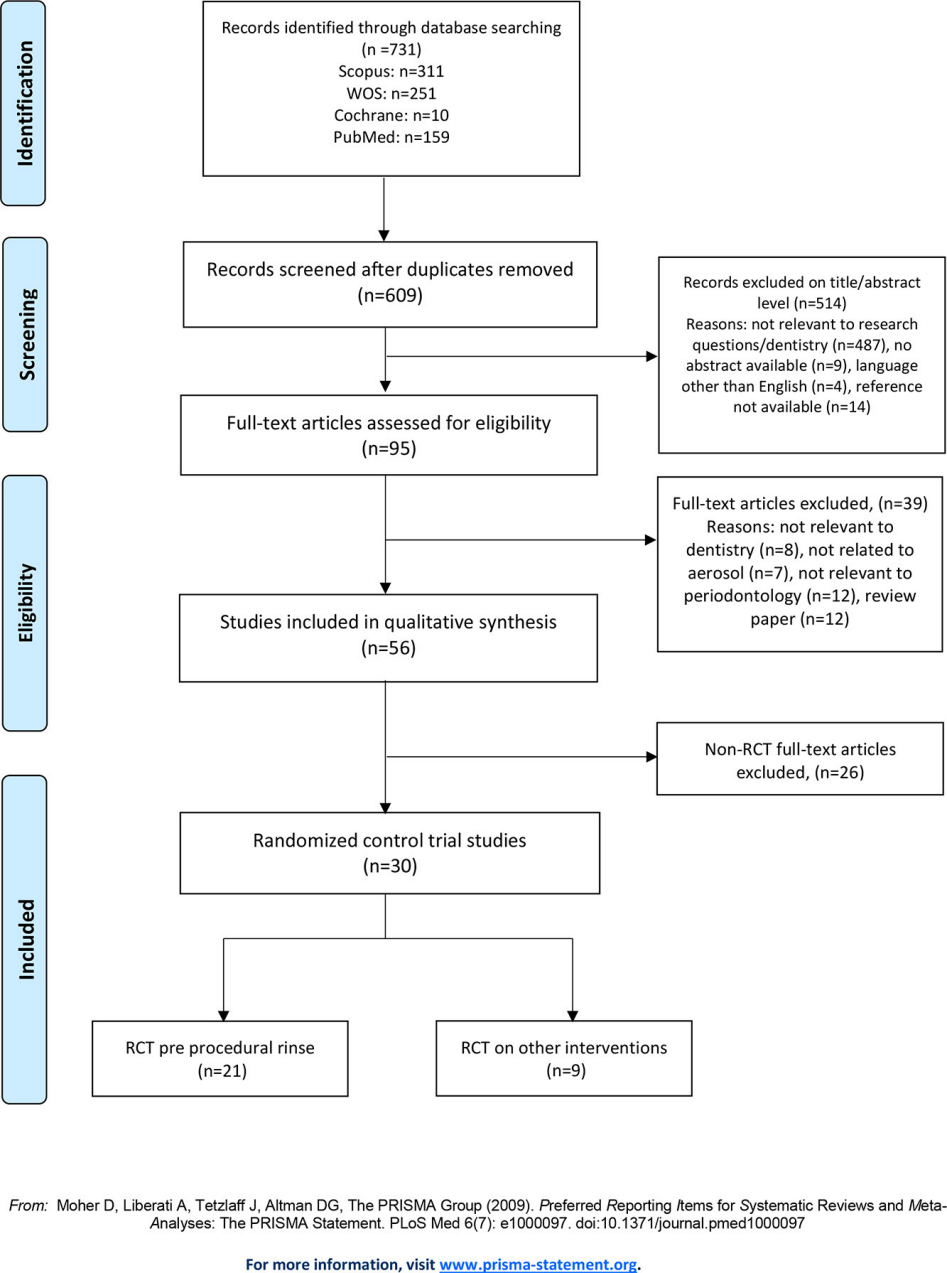
Summary of the selection process for systematic review.

The initial search yielded 731 articles across four databases. After removal of duplicates, the “title and abstract” of 609 articles were assessed, identifying 95 “potentially effective” studies (Agreement between assessors was high, **K** = 0.96). The full texts of potentially effective studies were then assessed, and 56 studies were identified as “effective studies” (Agreement between assessors was high **K** = 0.88). No additional studies were identified through “reference linkage” and among the effective studies 30 randomized control trial (RCT) studies were identified, 21 with a specific focus on pre-procedural rinsing (21–41) (Figure 1).

### Characteristics of the Studies

The study characteristics and periodontal procedures undertaken to generate aerosol in the studies included in this review are described in Table 1. Studies were published between 1992 and 2020. Approximately a quarter (6/21) were split-mouth design RCTs (21–25), 13 were full-mouth design (27–40), and one did not provide details of study design (41). The vast majority were parallel-arm TCTs (19/21) and two were RCTs of cross-over design. Over a third of studies (8/21) were identified as double-blinded interventions, and one claimed to be a single-blind study. The total number of participants among the studies was 984, ranging from 18 to 120 among studies, and of varying ages (18 to 70 years old). Participation was high among studies, except in one study where a subject received/ initiated antibiotics during the course of the study (36).

**Table 1:** Pre-procedural rinse study characteristics (Ctd).

**No**.	**References**	**Design**	**Subject size (N)**	**Pre-rinse intervention groups**	**Additional intervention group**	**Control group**	**Duration of rinse and interval before procedure**	**Periodontal procedure**
1	Devker et al. (21)	Split mouth, parallel	90 patients, 30 per group 18 - 45y/o	0.2% CHX	Scaling with HVE (140mmHg), CHX + scaling with HVE	No CHX pre-rinse/no HVE/no combination	2 min pre-rinse 10 ml *interval:NA*	Piezoelectric ultrasonic, 10 min scaling
2	Sawhney et al. (22)	Split mouth, parallel	60 patients, 20 per group 25–54y/o	0.2% CHX (1:1 in water), antiseptic (*contents: NA*) (1:1 in water)	With / without HVE	Water	2 x 30 sec pre-rinse 15ml CHX / 20ml antiseptic, *interval:NA*	Piezoelectric ultrasonic, *duration: NA*
3	Fine et al. (23)	Split mouth, crossover, double blind	18 patients, 9 per group *age: NA*	Antiseptic mouthwash (*contents and concentration: NA*)		5% hydro alcohol	30 sec pre-rinse 20 ml *interval:NA*	Magnetostrictive (Cavitron) ultrasonic, 5 min scaling
4	Saini (24)	Split mouth, parallel, double blind, placebo controlled	120 patients, 40 per group 18 - 55y/o	Chlorine dioxide, 0.2% CHX		Water	1 min pre-rinse, 10 ml *interval:NA*	Piezoelectric ultrasonic 10 min scaling, power medium 15ml/min flow
5	Fine et al. (25)	Split mouth cross over, double blind, repeated at 1 week after	18 patients, 9 per group >18y/o	Antiseptic mouthwash (*contents and concentration: NA*)		5% hydro alcohol	30 sec pre-rinse, 20 ml, 10 min before scaling	Ultrasonic, 10 min scaling
6	Narayana et al. (26)	Split mouth parallel	45 patients, 15 per group *age: NA*	0.12% CHX	With HVE (30 - 40 psikg/cm2)	No rinse, no HVE	30 sec pre-rinse 10 ml *interval:NA*	Piezoelectric (EMS) ultrasonic, 20 min scaling
7	Rajachandrasekaran et al. (27)	Full mouth, parallel	50 patients, 25 per group 20 - 50y/o	Herbal oral rinse (*contents and concentration: NA*)		0.12% CHX	1 min pre-rinse 15 ml 10 min before scaling	Magnetostrictive (Cavitron Bobcat Pro) ultrasonic, 10 min scaling
8	Shetty et al. (28)	Full mouth, parallel	60 patients, 20 per grp 25 - 45y/o	0.2% CHX, Tea tree oil (*concentration: NA*)		Distilled water	*Duration: NA* 10 ml 2 min before scaling	Ultrasonic 10 min scaling
9	Santos et al. (29)	Full mouth, parallel	23 patients, 23 per group 10-40y/o	0.12% CHX		Distilled water	1 min pre-rinse, 15 ml 10 min before prophylaxis	Jet hand I, sodium bicarbonate, 4 min polishing
10	Paul et al. (30)	Full mouth, parallel	60 patients, 20 per group 18 - 35y/o	0.2% CHX, 1% Povidone iodine, 94.5% aloe vera extract		None	1 min pre-rinse, *volume: NA* 10 min before scaling	Piezoelectric ultrasonic, 20 min scaling
11	Kaur et al. (31)	Full mouth, parallel, double blind	60 patients, 20 per group 20 - 50yold	0.2% CHX, 1% Povidone iodine	Irrigation with ozone(0.082mg/h)	None	1 min pre-rinse, *volume: NA interval:NA*	Ultrasonics, 10 min scaling
12	Retamal-Valdes et al. (32)	Full mouth, parallel, single blind	60 patients, 15 per group 18 - 70y/o	0.075% CPC + 0.28% Zn lactate + 0.05% NaF, 0.12% CHX + 10% alcohol		Water, no rinsing	1 min pre-rinse, 20 ml *interval: NA*	Magnetostrictive ultrasonic, 10 min scaling, frequency 25k Hz, power <50%
13	Reddy et al. (33)	Full mouth, parallel	30 patients, 10 per group *age: NA*	Non-tempered CHX 0.2%, tempered CHX 0.2%		Sterile water	1 min pre-rinse, *volume: NA interval:NA*	Ultrasonic scaling *duration: NA*
14	Gupta et al. (34)	Full mouth, parallel, double blind, placebo controlled	24 patients, 8 per group 25 - 55y/o	0.2% CHX, herbal extracts (*contents and concentration: NA*)		Water	1 min pre-rinse, 10 ml, 10min before scaling	Piezoelectric ultrasonic, 30 min scaling
15	Joshi et al. (35)	Full mouth, parallel, double blind	40 patients, 10 per group mean age 32.5	0.05% CPC (47°C), 0.2% CHX (47°C), 0.05% CPC (18°C), 0.2% CHX (18°C)			1 min pre-rinse 10 ml 10 min before scaling	Ultrasonic, 30 min scaling
16	Fine et al. (36)	Full mouth, crossover, double blind	18 patients, (1 initiated antibiotic, data excluded at the end) *per group: NA age:NA*	Antiseptic mouthwash (*contents and concentration: NA*)		5% hydro alcohol	30 s pre-rinse 20 ml 40 min before scaling	Magnetostrictive (Cavitron 3000) ultrasonic, 5 min scaling at each phase
17	Logothetis et al. (37)	Full mouth, parallel	18 patients 6 per group 25–54y/o	0.12% CHX, EO (*contents and concentration: NA*)		Distilled water	2 x 30 s pre-rinse 15 cc each rinse 10 min before air polishing	Polishing device, 3 min polishing
18	Feres et al. (38)	Full mouth, parallel, double blind, placebo controlled	60 patients, 15 per group 30 - 70y/o	0.05% CPC, 0.12% CHX		Water, no rinsing	1 min pre-rinse 15 ml *interval:NA*	Magnetostrictive (Cavitron Select) ultrasonic, 10 min scaling
19	Mohan et al. (39)	Full mouth parallel	20 patients, 10 per group 25 - 40y/o	0.2% CHX		Saline	1 min pre-rinse *volume: NA interval:NA*	Ultrasonic scaling *duration: NA*
20	Swaminathan et al. (40)	Full mouth, parallel	30 patients 10 per group 18–50y/o	0.2% CHX, herbal rinse (*concentration: NA*)		Saline	60 s pre-rinse 15 ml *interval:NA*	Ultrasonic scaling *duration: NA*
21	Serban et al. (41)	*NA*	80 patients, 40 per group 20–65y/o	0.1% CHX		Sterile water	*Duration: NA volume: NA interval:NA*	Ultrasonic scaling *duration: NA*

*HVE, high volume evacuator; CHX, chlorhexidine; CPC, cetylpyridinium chloride; PI, povidone iodine, EO, essential oil; NA, information not available inarticles*.

In eleven studies there were three experimental groups; nine studies had two or four experimental groups. One study had all participants as their own controls (41). Of the 21 RCTs, 18 used chlorhexidine (CHX)-containing mouth rinse as either a test (21, 22, 24, 26, 28–41) or as a positive control group (27). Other agents tested were novel antiseptics (four studies) (22, 23, 25, 36), herbal essential oils, EO (two studies) (28, 37), cetylpyridinium chloride, CPC (three studies) (32, 35, 38), povidone iodine (two studies) (30, 31), chlorine dioxide (one study) (24), aloe vera (one study) (30), and herbal extract (two studies) (34, 40). Controls used were saline, sterile water, distilled water, hydro alcohol, or no rinse at all.

Apart from using pre-rinses, some studies added other interventions to examine the impact on reducing bacterial load, namely the use of high-volume evacuation, HVE (three studies) (21, 22, 26) and irrigation using ozone (one study) (31). The protocol for pre-procedural rinsing varied: participants were instructed to rinse between 30 s and 2 min, the amount of mouth rinse used range between 10–20 ml, and participants waited between 2 and 40 min before they were given periodontal prophylaxis. Most studies (19/21) used ultrasonic scaling as the periodontal prophylaxis procedure, and two studies used polishing devices (29, 37). The duration of the periodontal prophylaxis ranged from 3 to 30 min.

### Effectiveness of Pre-procedural Rinse

Among the 21 studies, the majority (95.2%, 20) assessed bacterial contamination for aerosols (Table 2). For the most part, the key outcome measured was bacterial count expressed as colony forming units (cfu) on blood agar plates; however, incubation protocol differed among studies. One study assessed bacterial count per ml of blood, from blood drawn from the antecubital fossa (23); another study, in addition to aerosol bacterial contamination, assessed bacterial contamination from salivary samples (40). The sites of data collection differed among studies in terms of number of samples obtained, position, direction, and distance from subjects' mouth. However, there were no notable differences in the reduction of cfu from the aspects of periodontal prophylaxis devices used nor location of aerosol sampling collection from these studies. Approximately half of the studies (52.4%, 11/21) obtained the sample at or near the operator and dental assistant. Mostly, CHX rinse were tested (80.9%, 17/21), with various concentrations and volumes. Among studies comparing CHX with other agents (71.4%, 15/21), the effectiveness of CHX over other agents was evident, with more than half of the studies (7/15) reporting over a 70% reduction in cfu.

**Table 2:** Pre-procedural rinse study main findings (Ctd).

**No**.	**References**	**Outcome (unit)**	**Microbiological evaluation**	**Site of collection**	**Significant results (*p* values)**	**% of reduction of cfu**
1	Devker et al. (21)	Bacterial count (cfu)	Bacterial contamination in aerosol, cultured on blood agar, incubated at 37°C for 24 h,	From patient's mouth: 1. 6in - operator's nose, 2. 6in - assistant's nose, 3. 12in - patient's chest, 4. 36in - patient's right side	Significant cfu count/reduction: - in all groups, in all location (Student's paired t-test, - *p* < 0.01), - in HVE alone was better than CHX alone, - in combine CHX + HVE was better than CHX or HVE alone	Mean cfu reduction between CHX - no CHX, HVE - no HVE, CHX+HVE - scaling only at: - operator's nose: 59.2%, 83.2%, 88.1% - assistant's nose: 60.7%, 81.6%, 87.7% - patient's chest: 55.9%, 83.1%, 87.9% - patient's right side: 48.0%, 65.0%, 79.3%
2	Sawhney et al. (22)	Bacterial count (cfu)	Bacterial contamination in aerosol, cultured on blood agar plates incubated at 37°C for 24 h	1. Patient's chest 2. Dental unit tray, 3. 6in away from patients' mouths	Significant cfu count/reduction: - in all groups (ANOVA, *p* < 0.05) - between CHX-water (Student's t-test, *p* = 0.001) and CHX-Listerine (Student's *t*-test, *p* = 0.025)	Mean aerobic cfu reduction between CHX, antiseptic and water: - with use of HVE: 95%, 70%, 40% - without use of HVE: 65%, 45%, 20%
3	Fine et al. (23)	Bacterial count per ml blood (cfu/ml)	Blood drawn from antecubital fossa then incubated on agar plates at 37°C for 24 h (aerobically), or 5 days (anaerobically)		Significant cfu count/reduction between antiseptic and control both aerobic and anaerobic colonies (*p* = 0.00001)	Mean cfu reduction between antiseptic-control for: - aerobic: 92.3% - anaerobic: 87.8%
4	Saini (24)	Bacterial count (cfu)	Bacterial contamination in aerosol, cultured on blood agar, incubated at 37°C for 48 h	From patient's mouth: 1. 1 feet - patient's chest 2. 1 feet - operator 3. 1 feet - assistant 4. 2 feet - 12 o'clock 5. 8 feet - 6 o'clock	Significant cfu count/reduction: - in ClO2 and CHX compared to water (ANOVA, *p* < 0.001) in all positions, - highest at patient's front, and almost the same in all other areas (ANOVA, *p* < 0.001)	Mean cfu reduction between water, ClO2, CHX at: - patient's chest: 2.1%, 85.4%, 88.0% - operator position: 2.1%, 85.7%, 87.7% - assistant position: 2.3%, 85.3%, 88.1% - 12 o'clock: 3.8%, 85.7%, 87.6% - 6 o'clock: 3.4%, 89.2%, 92.8%
5	Fine et al. (25)	Bacterial count (cfu)	Bacterial contamination in aerosol, cultured on blood agar, incubated at 37°C for 24–72 h	From patient's mouth: 1. 1 feet - patient's chest 2. 1 feet - operator's chest 3. 1 feet - assistant' chest 4. 2 feet - 12 o'clock 5. 8 feet - 6 o'clock	Significant cfu count/reduction: - in CHX compared to control (Student's *t* test, *p* < 0.001)	Mean cfu reduction between CHX and control at was 94.1% and 33.9%
6	Narayana et al. (26)	Bacterial count (cfu)	Bacterial contamination in aerosol, cultured on on blood agar incubated at 37°C for 48 h	Left of patient (between patient and assistant)	Significant cfu count/reduction: - in CHX pre-rinse grou*p* compared to no rinse (ANOVA, *p* < 0.001) - when HVE was used compared to no HVE (ANOVA, *p* < 0.001) - when both CHX and HVE were used (ANOVA, *p* < 0.001)	Mean cfu reduction between test-control groups for use of: - CHX: 61.4% - HVE: 67.0% - combination of CHX+HVE: 86.0%
7	Rajachandrasekaran et al. (27)	Bacterial count (cfu)	Bacterial contamination in aerosol, cultured on MeReSa with supplements agar plates, incubated at 37°C for 48 h	From patient's mouth: 1. 2 feet - patient's right 2. 2 feet - behind patient 3. 2 feet - patient's left 4. 3 feet - patient's right 5. 3 feet - patient's left 6. 5 feet - patient's right 7. 5 feet - patient's left 8. 9 feet	Significant cfu count/reduction: - in CHX (control) compared to herbal for MRSA (One way ANOVA, *p* = 0.0001) - in herbal group compared to CHX for MRSA colony compared to Actinobacter in all 8 locations (One way ANOVA, p = 0.0001)	Mean cfu reduction between herbal - CHX: - for MRSA: 55.6% - for Actinobacter: 17.5%
8	Shetty et al. (28)	Bacterial count (cfu)	Bacterial contamination in aerosol, cultured on Trypticase soy agar plates	1.6in - operator's nose 2. 6in - dental assistant's nose 3. 12in - patient's chest level	Significant cfu count/reduction: - between CHX-water, CHX-TTO, TTO-water, (Kruskal-Wallis, Mann-Whitney U, p < 0.001)	Mean cfu reduction between CHX-water, TTO-water, CHX-TTO at all positions were 20.8%, 6.7%, 27.7%
9	Santos et al. (29)	Bacterial count (cfu)	Bacterial contamination in aerosol, cultured on Brain heart infusion (BHI) agar, incubated at 37°C, 48 h	1. operator's forehead 2. 10cm from operator's mouth (vertical downward) 3. 15cm from patient's mouth - patient's chest	Significant cfu count/reduction: - between interval T1 - T2 for both CHX and water (Wilconxon test comparing within groups; p < 0.001) - in al 3 positions (Kruskal-Wallis): i- clinicians forehead: p = 0.0074 ii- clinician's chest: p = 0.0051 iii- patient's chest: p = 0.0035	Mean cfu reduction following CHX rinse at 1month at: - operator's forehead: 36.1% - operator's chest: 35.8% - patient's chest: 40.5%
10	Paul et al. (30)	Bacterial count (cfu)	Bacterial contamination in aerosol, cultured on blood agar, incubated at 37°C for 48 h	From patient's mouth: 1. 12in - patient's chest 2. 12in - operator's chest	Significant cfu count/reduction: - in all groups, in both location (one-way ANOVA, p = 0.001) - highest in CHX, AV then PVO-I (independent *t* test, p = 0.001) - between CXH - PVP-I and PVP-I - AV (ANOVA, *post hoc* comparison, p = 0.001)	Mean cfu reduction between CHX - PVP-I, CHX - AV and PVP-I - AV at: - operator's chest: 69.1%, 9.3%, 66.0% - patient's chest: 60.4%, 8.3%, 56.8%
11	Kaur et al. (31)	Bacterial count (cfu)	Bacterial contamination in aerosol, cultured on blood agar, incubated at 37°C for 48h	1. operator's chest, 2. 9feet behind patient's head	Significant cfu count/reduction: - in all groups, in all locations (paired t test, *post-hoc* Tukey's test) for aerobic (p < 0.01) and anaerobic bacteria (*p* < 0.001) - for aerobic bacteria at patient's chest between CHX-PI and PI-Ozone (*p* < 0.01) - for anaerobic at patient's chest between CHX-Ozone (*p* < 0.05)	Aerobic cfu reduction between CHX, PI, Ozone at: - operator's mask: 57%, 54%, 47% - patient's chest: 35%, 37% 29% - behind patient: 36%, 47%, 29% - anaerobic cfu reduction between CHX, PI, ozone at: - patient's chest: 43%, 36%, 35% - behind patient: 44%, 32%, 38%
12	Retamal-Valdes et al. (32)	Bacterial count (cfu)	Bacterial contamination in aerosol, cultured on enriched TSA blood agar, incubated at 37°C for 72 h	1. support board in front of patient 2. operator's forehead 3. patient's chest	Significant cfu count/reduction: - in CPC+Zn+F and CHX compared to water and those who did not rinse (Kruskall-Wallis and Dunn tests, p < 0.05) at operator's forehead and patient's chest	Mean cfu reduction between CPC+Zn+F / CHX - no rinsing at: - all areas: 70%, 77% - operator's forehead: 89%, 94% - patient's chest: 55%, 60% - support board: 70%, 81% Mean cfu reduction between CPC+Zn+F / CHX - water at: - all areas: 61%, 70% - operator's forehead: 78%, 87% - patient's chest: 55%, 60% - support board: 59%, 75%
13	Reddy et al. (33)	Bacterial count (cfu)	Bacterial contamination in aerosol, cultured on blood agar, incubated at 37°Cfor 48 h	4 feet from patient's mouth: 1. 3 o'clock 2. 6 o'clock 3. 12 o'clock	Significant cfu count/reduction: - in tempered and non-tempered CHX compared to water (ANOVA, *p* < 0.001) - in all positions as a cumulative data	Mean cfu reduction between water, non-tempered, tempered CHX at: 19.3%, 83.2%, 90.0%
14	Gupta et al. (34)	Bacterial count (cfu)	Bacterial contamination in aerosol, cultured on blood agar, incubated at 37°C for 48 h	1. patient's chest 2. operator's chest 3. assistant's chest	Significant cfu count/reduction: - among all 3 groups (ANOVA, *p* < 0.001) in all 3 locations - CHX significantly reduced cfu compared to herbal mouthwash (independent t test, *p* < 0.001) in all 3 locations	Mean cfu reduction between CHX - water, herbal - water, CHX - herbal at: - patient's chest: 71.3%, 38.4%, 35.2% - operator's chest: 71.6%, 35.0%, 36.6% - assistant's chest: 16.1%, 6.9%, 9.3%
15	Joshi et al. (35)	Bacterial count (cfu)	Bacterial contamination in aerosol, cultured on blood agar plates, *details of incubation: NA*	From patient's mouth: 1. 12in - neck of patient 2. 12in - operator's chest 3. 12in - assistant's chest	Significant cfu count/reduction: - patient's chest had highest contamination (*p* = 0.02) - between cold CPC - cold CHX (unpaired *t*-tests, *p* = 0.0284) - equally in CPC and CHX (ANCOVA, *p* < 0.001) - greater in warm CPC and CHX (*p* < 0.001)	Mean cfu reduction CPC - CHX at: - all positions: 0.68% - 21.8% (not significant) - patient: warm CPC - cold CPC 26.3%, warm CHX - cold CHX 31.8% - operator: warm CPC - cold CPC 18.2%, warm CHX - cold CHX 31.4% - assistant: warm CPC - cold CPC 13.0%, warm CHX - cold CHX 20.9%
16	Fine et al. (36)	Bacterial count (cfu)	Bacterial contamination in aerosol, cultured on enriched soy agar, incubated at 37°C for 24–72 h	2 in from patient's mouth	Significant cfu reduction between antiseptic and water (*p* = 0.0001)	Mean cfu reduction between test - control was 93.6% and 32.1%
17	Logothetis et al. (37)	Bacterial count (cfu)	Bacterial contamination in aerosol, cultured on blood agar plates incubated at 37°C for 48h	From patient's mouth: 1. operator's mask 2. 2 feet - assistant 3. 3 feet - 12 o'clock 4. 3 feet - 9 o'clock 5. 3 feet - 3 o'clock 6. 5 feet 8in - 4 o'clock 7. 6 feet - 6 o'clock 8. 9 feet - 6 o'clock	Significant cfu reduction: - in all locations by CHX compared to others (*p* = 0.001)	Mean cfu reduction between CHX-water, EO-water and CHX-EO at: - operator's mask: 69%, 1%, 70% - 2 feet - assistant: 60%, 7%, 67% - 3 feet - 12 o'clock: 43%, 8%, 51% - 3 feet - 9 o'clock: 42%, 0%, 42% - 3 feet - 3 o'clock: 42%, 0%, 42% - 5 feet 8in - 4 o'clock: 35%,−5%, 30% - 6 feet - 6 o'clock: 30%,−5%, 25% - 9 feet - 6 o'clock: 25%,−5%, 20%
18	Feres et al. (38)	Bacterial count (cfu)	Bacterial contamination in aerosol, cultured on blood agar incubated with 10% CO2 at 37°C for 72 h	From patient's mouth: 1. 12in - clinician's forehead 2. 12in - support board 3. 12in - patient's chest	Significant cfu count/reduction: - in CPC and CHX compared to water and no rinsing - equally in CPC and CHX in all locations (Kruskal-Wallis and Mann-Whitney *U* tests, *p* < 0.05)	Mean cfu reduction between CPC - water / no rinsing and CHX - water / no rinsing at: - all: CPC: 68% / 77%, CHX: 70% / 78% - operator: CPC: 79% / 78%, CHX: 73% / 72% - board: CPC: 79% / 82%, CHX: 76% / 79% patient: CPC: 61% / 65%, CHX: 66% / 78%
19	Mohan et al. (39)	Bacterial count (cfu)	Bacterial contamination in aerosol, cultured on blood agar incubated at 37°C for 24 h	3 feet from patient (6 o'clock)	Significant cfu count/reduction: - within CHX (*p* = 0.0049) - between CHX and saline (*p* = 0.0037)	Mean cfu reduction between CHX - saline was 66.6% and 2.0%
20	Swaminathan et al. (40)	Bacterial count (cfu) in saliva and aerosol	Bacterial contamination in aerosol, cultured on BHI agar incubated anaerobically at 37°C for 24 h	From patient's mouth: 1. 1 feet 2. 2 feet 3. 3 feet	Significant cfu count/reduction in: - aerosol at 3feet and saliva in all locations (Kruskal-Wallis, *p* < 0.001) - between CHX-saline, herbal-saline, CHX-herbal (*p* < 0.001)	Mean cfu reduction between CHX - saline and herbal – saline at: In aerosol: - 1 feet: 56.2%, 24.9% - 2 feet: 50.6%, 17.5% - 3 feet: 62.7%, 37.7% In saliva: - saline / CHX / herbal:−182% / 64.7% / 0.4% - CHX – saline: 103.0%, - herbal – saline: 47.7%
21	Serban et al. (41)	Total number of bacteria (cfu/m3).	Bacterial contamination in aerosol, cultured on blood agar, *details of incubation: NA*	Dentist's mask	Significant cfu count/reduction: - in CHX compared to water for bacteria (*p* < 0.001) and haemolytic bacteria (*p* < 0.001)	Mean cfu reduction between CHX - water for: - bacteria: 83.2% - haemolytic bacteria: 74.8%

*HVE, high volume evacuator; CHX, chlorhexidine; CPC, cetylpyridinium chloride; PI, povidone iodine; EO, essential oil; NA, information not available in articles*.

### Assessment of Bias

**Figure 2:**
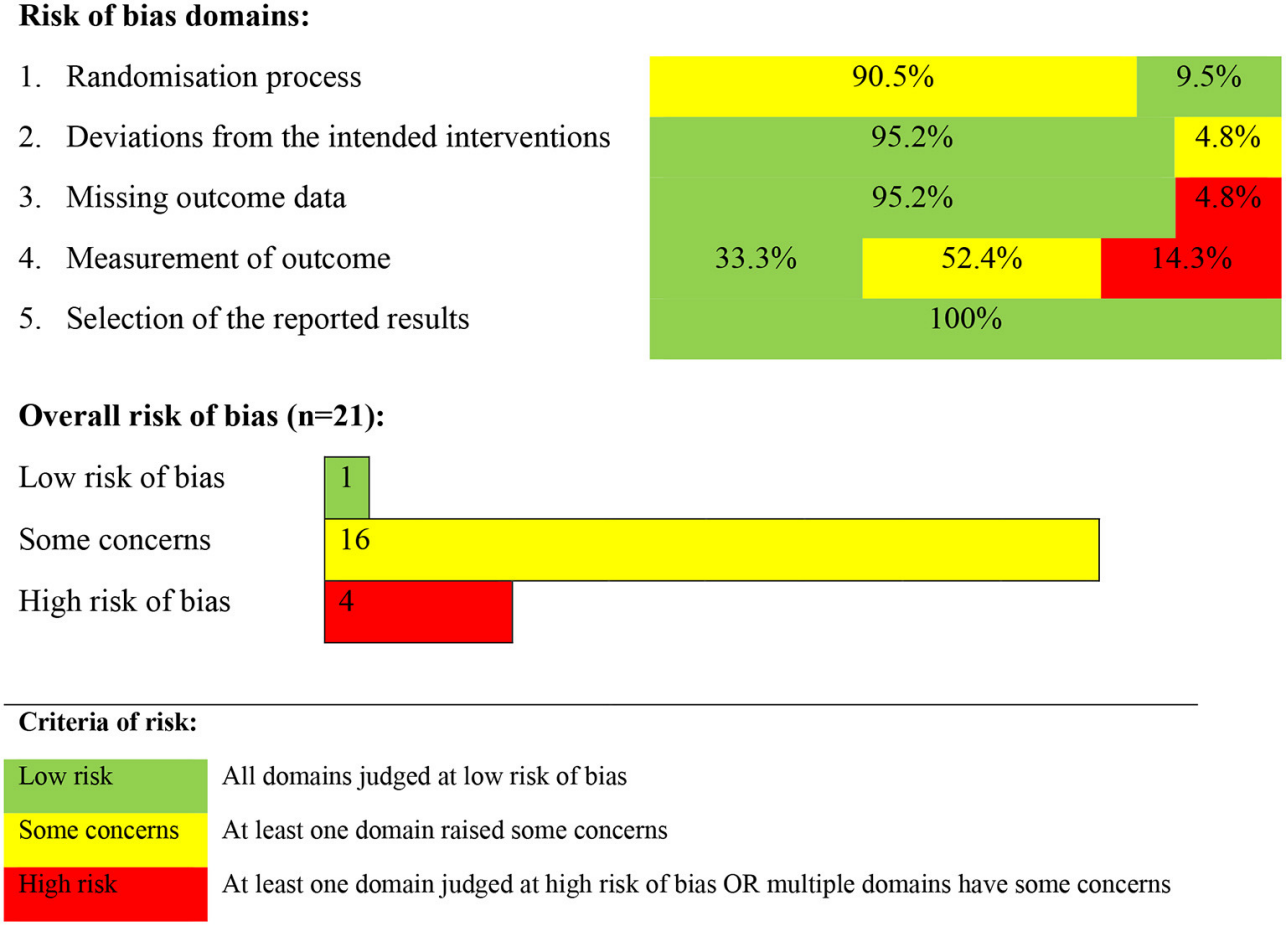
Risk of bias criteria for the literature studied.

Risk of bias varied among studies, Figure 2. For the most part (90.5% of studies), there was of unclear risk of bias in terms of the randomization process (domain 1) with 9.5% being of low risk of bias. There was generally a low risk of bias in terms of deviation from the intended interventions (95.2% of studies, domain 2) and the remaining were of questionable risk of bias (4.8%). There was a high risk of bias regarding missing outcome data in approximately 1 in 20 studies (4.8%), although 95.2% of studies in this regard were of low risk of bias (95.2%). For a third of studies (33.3%) there was a low risk of bias regarding the measurement of outcome; approximately half (52.4%) had an unclear risk of bias and 14.3% were of high risk of bias. In terms of selection of reported results, all studies were of low risk of bias.

## Discussion

As the number of fatalities and morbidities due to COVID-19 continues to rise it is important to acknowledge that oral health remains an integral element of overall health and well-being (42). Despite the uncertainties associated with the exact characteristics for SARS-Cov-2 transmission in dental settings, the dental team and administration must ensure safety of all personnel and patients, as well as other related parties involved in providing dental care to the public, such as cleaning and services. Reducing contamination of aerosol produced in dental clinics is a simple way to minimize and prevent cross-infections and has been increasingly reviewed during this pandemic (8, 43). The majority of these reviews focus on recommendations for general precautions in dental practice, but do not emphasize the aerosol contamination from periodontal prophylaxis, as one of the most common AGPs in dental clinics.

Our review is limited to aerosol contamination. Although our search was focused on microbial contamination in dental aerosol, there was no literature found on virus, which would provide more insight on possible COVID-19 cross-contamination in dental clinics. A majority of these studies included the use of CHX as the main antiseptic agent to reduce aerosol contamination, as it is a well-established antimicrobial agent used in dentistry. CPC has also been widely tested in anti-plaque and anti-gingivitis studies (44, 45) as well as infection control in the dental clinic for disinfection of hands and surfaces (46, 47). Its potential to reduce microbial load after dental scaling is promising and, in this review, CPC had been included in three of the studies.

The key outcome measure among studies was bacterial contamination, which is predominantly related to associated aerosol contaminations. Given the importance of viruses such as COVID-19, there is a need to investigate the effectiveness of mouth rinses on viral counts. The lack of *in vivo* viricidal studies in part relates to the challenges and associated risk in culturing the virus (17). *In vitro* studies have shown chlorhexidine (0.12%) to be effective against most viruses, in as little as 30 s (48). Difference in effectiveness is thought to be due to physical/chemical structures of viruses' envelopes. In addition, several mouth rinses have been shown to be effective against standard strains of fungi *in vitro* (49). Whilst evidence of aerosol contamination and evidence of growth from such does suggest, at least theoretically, the risk of transmission, evidence from blood, saliva, or other samples can confirm actual transmission and potentially the reduction in risk following use of mouth rinses (50, 51). All studies demonstrated some degree of effectiveness of the mouth rinses, although their effectiveness varied widely. This is in part related to differences in study design (i.e., split mouth vs. subject level randomisation), differences in mouth rinses agent, differences in concentration among the same agent, differences in number and where assessments were conducted (i.e., distance from patient's mouth), and the use of other preventive measures (such as use of high-volume suctions). Such heterogeneity among studies precludes the ability to perform a meta-analysis. Nonetheless, the evidence suggests that mouth rinses are effective in reducing bacterial contamination and there is a need to consider their role in preventing viral infections, such as COVID-19.

A recent publication by Meister et al. (52) reported potential *in vitro* antiviral efficacy of some commercially available oral rinses against strains of SARS-CoV-2 when exposed within 30 s in saliva. Reasonably, strains of the virus reacted differently in many levels of susceptibility depending on the formulation prepared and contents of active ingredients in these rinses. Nevertheless, this finding may mark a turning point in efforts to lower the transmission of viruses, such as COVID-19, through use of oral rinses in the near future. Also noteworthy are the new trials being conducted to study the efficacy of oral mouthwashes on COVID-19 patients (https://clinicaltrials.ucsf.edu/trial/NCT04409873, https://clinicaltrials.gov/ct2/show/NCT04341688) that could help provide safe alternatives to dental practices and healthcare for the public at large.

Continuous periodontal care for periodontitis patients is of the utmost importance and must be done in a timely manner to prevent progression and reinfection of the disease, especially among high-risk individuals (53–56). Progression and reinfection of periodontal pockets does not only have implications for oral health, but also systemic health, given the bidirectional link between periodontal disease and common non-communicable diseases such as diabetes mellitus, rheumatoid arthritis, and hypertension, and those who are immune suppressed. The treatment needs of the patients should not be hampered during this pandemic, but instead should be further prioritized, as there are restrictions of movement, limited access to dental care, and fear of cross-infections from dental clinics. Resolving this issue would offer more benefits than containing the progression of periodontal disease in the population, as it would also improve the quality of life of patients (57, 58) with long-term supportive care (55).

Pre-procedural rinsing for clinical dental procedures has long been advocated for and, during the current pandemic, its importance to practice is more relevant than ever. A number of acceptable quality RCTs of pre-procedural mouth rinsing have been conducted. The focus has been on bacterial contamination rather than viral or fungal assay. A range of different pre-procedural mouth rinses have been tested (mostly chlorhexidine), of varying concentrations and amounts, and for dental procedures of different durations and using different techniques with and without additional means. We acknowledge that these systematic review findings do not differ much from the previous reviews, nevertheless it emphasizes on the importance of pre-rinsing particularly in periodontal prophylaxis procedures. This is especially important during the current pandemic where transmission bacterial-borne diseases could well be avoided by reducing the risk on dental personnel as well as the patients. This also supports the need to include the pre-rinsing procedure for dental patients as one of the mandatory SOP in current practice.

## Conclusion

Our systematic review found no remarkable new evidence on the effectiveness of pre-procedural rinse in periodontal prophylaxis, but it highlights the importance of the procedure to be given a mandatory emphasis in the current pandemic SOP in dental clinics. Pre-procedural rinsing for 30 s to 2 min with selected antimicrobial solutions compared to water or no rinsing were found to effectively reduce aerosol contamination in periodontal prophylaxis on dental patients. There is evidence that chlorhexidine (either 0.12 or 0.2%) is an effective antimicrobial solution for this purpose. The use of HVE during the procedure also helps to reduce the aerosol contamination.

## Data Availability Statement

The raw data supporting the conclusions of this article will be made available by the authors, without undue reservation.

## Author Contributions

CM, TM-D, and SM-S: conceptualization. CM, TM-D, and SM-S: methodology. SM-S, HR, TM-D, and CM: analysis. SM-S, NS, and TM-D: writing—original draft preparation. SM-S, HR, TM-D, and CM: writing—review and editing. CM: supervision. All authors contributed to the article and approved the submitted version.

## Conflict of Interest

The authors declare that the research was conducted in the absence of any commercial or financial relationships that could be construed as a potential conflict of interest.
