# Supplementary material for: Effectiveness of Pre-procedural Mouth Rinses in Reducing Aerosol Contamination During Periodontal Prophylaxis: A Systematic Review
Source: Front Med (Lausanne). 2021 Jun 10;8:600769. doi: 10.3389/fmed.2021.600769 (PMC8222587; doi:10.3389/fmed.2021.600769)
Supplement: Supplementary file 1 [file Data_Sheet_1.docx]

**Appendix: Search strategy**

| **Database** | **Search string** | **Limits** |
| --- | --- | --- |
| Scopus | TITLE-ABS-KEY (aerosol* OR bioaerosol OR bio-aerosol* OR "bio aerosol*" ) AND ( "dental prophylaxis" OR "periodontal scaling" OR scaling OR "root planing" OR "debridement" OR "periodontal debridement" OR "dental polishing" OR "air polishing" OR "air abrasion" ) | **Language**: English **Subarea**: Medical, Dental, Immunology, Health, Nursing |
| MEDLINE | ((aerosol*[Title/Abstract]) OR (bioaerosol[Title/Abstract]) OR (bio-aerosol*[Title/Abstract]) OR ("bio aerosol*"[Title/Abstract])) AND (("dental prophylaxis"[Title/Abstract]) OR ("periodontal scaling"[Title/Abstract]) OR (scaling[Title/Abstract]) OR ("root planing"[Title/Abstract]) OR ("debridement"[Title/Abstract]) OR ("periodontal debridement"[Title/Abstract]) OR ("dental polishing"[Title/Abstract]) OR ("air polishing"[Title/Abstract]) OR ("air abrasion"[Title/Abstract])) | **Language**: English |
| Cochrane Library | aerosol in Title Abstract Keyword AND dental prophylaxis in Title Abstract Keyword AND air polishing | **Language**: English **Type of document**: Cochrane Reviews, Cochrane Protocols, Trials, Clinical Answers |
| Web of Science | TS=(( aerosol* OR bioaerosol OR bio-aerosol* OR "bio aerosol*") AND ("dental prophylaxis" OR "periodontal scaling" OR scaling OR "root planing" OR "debridement" OR "periodontal debridement" OR "dental polishing" OR "air polishing" OR "air abrasion"))) | **Language**: English **Research areas:** Dentistry, Oral Surgery and Medicine, Microbiology, Infectious Diseases, Research Experimental Medicine, Medical Informatics, Public Environmental Occupational Health, Virology, Immunology, Otorhinolaryngology, Health Care Sciences and Services, Legal Medicine, Emergency Medicine, Education and Educational Research |
